# An Update on Isolation Methods for Proteomic Studies of Extracellular Vesicles in Biofluids

**DOI:** 10.3390/molecules24193516

**Published:** 2019-09-27

**Authors:** Jing Li, Xianqing He, Yuanyuan Deng, Chenxi Yang

**Affiliations:** 1 School of Chemistry, Chemical Engineering and Life Science, Wuhan University of Technology, 122 luoshilu, Wuhan 430070, China; lij@whut.edu.cn (J.L.); 714769257@whut.edu.cn (X.H.); 2 School of Biological Science & Medical Engineering, Southeast University, No.2 Sipailou, Nanjing 210096, China; 213150742@seu.edu.cn

**Keywords:** extracellular vesicles, isolation methods, biofluid, proteomics, mass spectrometry

## Abstract

Extracellular vesicles (EVs) are lipid bilayer enclosed particles which present in almost all types of biofluids and contain specific proteins, lipids, and RNA. Increasing evidence has demonstrated the tremendous clinical potential of EVs as diagnostic and therapeutic tools, especially in biofluids, since they can be detected without invasive surgery. With the advanced mass spectrometry (MS), it is possible to decipher the protein content of EVs under different physiological and pathological conditions. Therefore, MS-based EV proteomic studies have grown rapidly in the past decade for biomarker discovery. This review focuses on the studies that isolate EVs from different biofluids and contain MS-based proteomic analysis. Literature published in the past decade (2009.1–2019.7) were selected and summarized with emphasis on isolation methods of EVs and MS analysis strategies, with the aim to give an overview of MS-based EV proteomic studies and provide a reference for future research.

## 1. Introduction

Although extracellular vesicles (EVs) were first described as ‘platelet dust’ in the late 1960s, it is now widely accepted that EVs are novel and important mediators for cellular communication by delivering bioactive molecules from donor to recipient cells [1,2]. Growing evidence has indicated that the cargo of EVs can reflect the content of their cells of origin and regulate physiological and pathological processes [3]. To date, EVs are considered as a novel source for biomarker discovery. With the benefits of liquid biopsy, analysis of EVs in biofluids has emerged as a promising diagnostic and monitoring tool for many diseases including cancer, neurodegenerative, kidney, and cardiovascular diseases [1,4,5].

EVs are membrane-enclosed particles that carry many bioactive molecules, including nucleic acids, proteins, and lipids, from their cells of origin. Based on their intracellular origin, EVs can be classified into three categories: exosomes, microvesicles (MVs), and apoptotic bodies. Exosomes are classically defined as the nanoparticles with sizes from 30–100 nm and formed by the fusion of multivesicular bodies with the plasma membranes; microvesicles, also called ectosomes, are usually described as the particles with sizes from 100–1000 nm and directly budded from the plasma membrane; apoptotic bodies (>1000 nm) are often considered as the particles that are released by apoptotic cells [6,7]. Despite apparent differences from their definition, it is difficult to differentiate the types of EVs after their release. It has been shown that the size of exosomes and microvesicles has a considerable overlap [7]. Currently, most of the isolation methods described in this review result in the mixed population of EVs. In addition to the physical heterogeneity, EVs are also highly heterogeneous in their cargo composition. Significant efforts have been made with the aim to comprehensively categorize EV subtypes, such as building an extensive and up–to–date database for EVs including ExoCarta, Vesiclepedia, and EVpedia [8,9,10,11]. However, consensus regarding the molecular markers to unambiguously distinguish the types of EVs remains to be a problem. Therefore, ‘extracellular vesicle’, which is suggested by the International Society of Extracellular Vesicles (ISEV), is used here for all the secreted vesicles [12].

**Figure 1:**
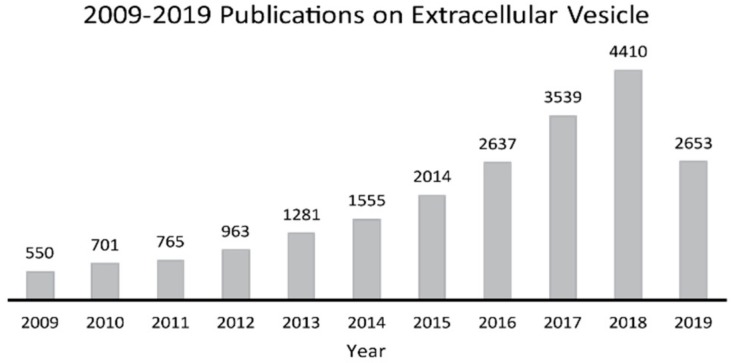
Publication trends on extracellular vesicle studies in the past decade (2009.1 to 2019.7). Publications were selected by searching the keyword “extracellular vesicle” in the Web of Science from the year of 2009.1 to 2019.7. x axis: year; y axis: number of publications.

Due to their tremendously diagnostic and therapeutic potential, EVs have gained increasing attention in the past decade, as shown by the number of publications (Figure 1). However, most of the studies focus on the nucleic acid content of EVs, such as microRNA or messenger RNA. With its improvements on sensitivity and high-throughput, mass spectrometry (MS) has become the fundamental technique of proteomics in recent years. Nowadays, MS has the capability to identify and characterize the protein content of EVs [6]. In the past decades, MS has been utilized to study EV proteome in various diseases, such as cancer and cardiovascular diseases [13,14]. This review will focus on publications within ten years that contain MS-based studies for EV proteins in human biofluids, such as urine, plasma, and saliva, rather than studies of EVs from laboratory animals or cell cultures and without any MS characterization. The references may be not comprehensive, but we try to highlight the recent improvements on isolation and MS strategies used in studies of EV proteome.

## 2. Isolation Strategies for Extracellular Vesicles in MS-Based Proteomic Studies

**Figure 2:**
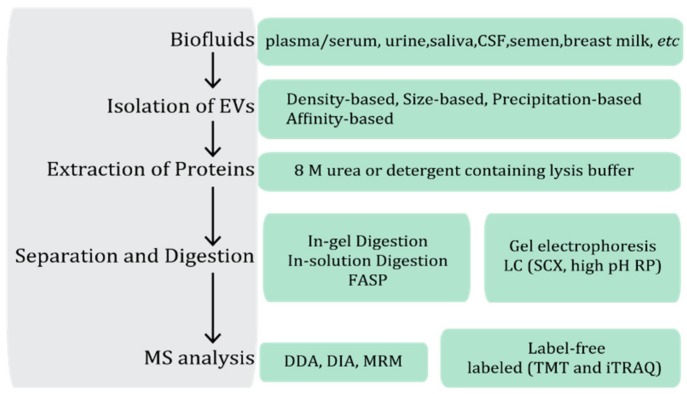
A general workflow of mass spectrometry (MS)-based proteomic extracellular vesicle (EV) study. EVs are firstly isolated from various biofluids, and EV proteins are extracted by adding detergent or non-detergent containing lysis buffer. The extracted EV proteins can be separated by gel electrophoresis and digested in-gel before MS analysis. Alternatively, digestion can be performed after protein extraction, and the generated peptides are either fractionated by liquid chromatography (LC) before MS analysis or directly subjected to MS analysis. The MS analysis can be conducted in data-dependent acquisition (DDA) or data-independent acquisition (DIA) for discovery EV studies or multiple reaction monitoring (MRM) for target EV studies. Differential expressed EV proteins also can be revealed by quantitative MS analysis via label-free or labeled quantitative proteomics. CSF: cerebrospinal fluid; FASP: filter aided sample preparation; SCX: strong cation exchange chromatography; RP: reverse phase chromatography; TMT: tandem mass tag; iTRAQ: isobaric tag for relative and absolute quantitation.

EVs in biofluids are several orders of magnitude lower than other abundant components, such as lipoprotein particles, protein aggregates, and soluble proteins, including albumin in blood and Tamm-Horsfall protein (THP) in urine, which could interfere with the characterization of EVs [15,16]. Thus, the isolation step is required for all EV studies. In a typical MS-based bottom-up proteomic workflow, an additional isolation step for EVs is applied before the protein extraction and digestion (Figure 2). The commonly used isolation methods are either through the physical property of EVs, such as density and size, or based on the chemical property of EVs, such as through interacting with surface proteins of EVs, to achieve isolation [15]. Even though microfluidics-based devices hold promising potential for rapid and efficient isolation of EVs from biofluids, their low processing capacity greatly limits the downstream analysis due to the insufficient amounts of proteins [17]. Hence, this review will discuss the isolation methods, which could provide successful downstream MS-based proteomic EV studies and give an update for the ten-year improvements on isolation methods which are used in MS-based workflow studies.

### 2.1. Sample Storage and Processing Conditions

Inappropriate storing and processing conditions can significantly affect the EV characteristics and recovery from biofluids, thus increasing pre-analytical variances or bringing artificial results. However, this aspect is not the focus of this review, and several comprehensive review or research papers have covered this topic [11,15,18,19,20]. Herein, some suggestions which are important and have been universally understood by the community are listed. In general, samples should be processed immediately after collection and in minimal waiting periods between each processing stages. Aliquots of samples are recommended in order to avoid multiple freezing–thawing cycles during whole processes. To obtain better EV recovery and preserve their characteristics in the biofluids, storing samples at −80 °C before EV isolation is important for long time storage [18,21,22,23]. However, one should be aware that there are no strict standards regarding sample storage and processing conditions for now. Most studies focus on the effects on concentration, size, RNA content, or some of the marker proteins of EVs under different conditions [18,21,24]. The comprehensive proteomic studies are still needed for evaluating the effects on protein content. In addition, each type of biofluid has special considerations which should be noticed before starting experiments.

### 2.2. Density-Based Isolation

**Figure 3:**
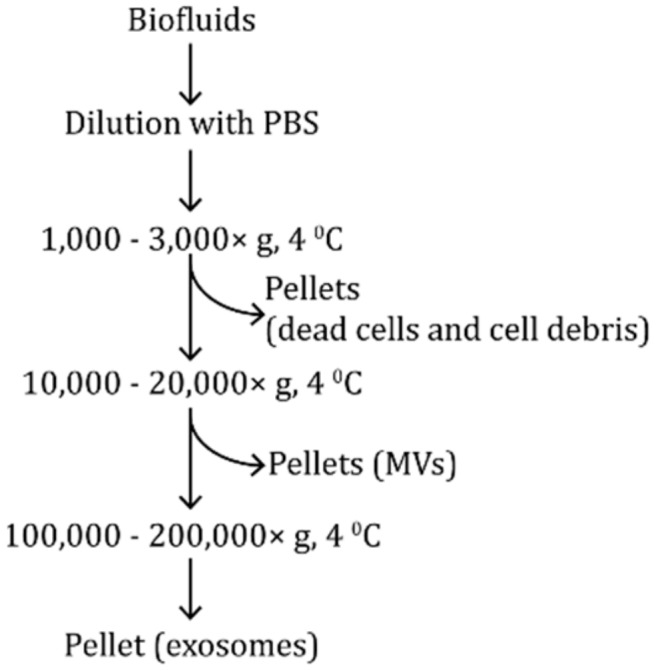
A basic differential ultracentrifugation (dUC) workflow for isolation of MVs and exosomes. Biofluids are diluted by phosphate-buffered saline (PBS) before centrifugation. Dead cells and cell debris are removed as pellets during the centrifugation at 1000–3000× *g*. Further centrifugation of supernatant at 10,000–20,000× *g* facilitates the isolation of MVs from exosomes. Finally, the recovery of exosomes is achieved by ultracentrifuging the 10,000–20,000× *g*-derived supernatant at 100,000–200,000× *g*.

Differential ultracentrifugation (dUC) as the current gold standard is the most commonly used isolation method of EVs. A recent worldwide survey of ISEV members has reported that 80% of EV isolation was conducted by dUC [25]. Biofluids typically contain a multicomponent mixture of particles that differ in sizes and densities, thus resulting in different sedimentation rates. During dUC, smaller particles can be isolated from larger ones according to their sedimentation rates by a successive increase of centrifugation forces and durations [26]. Although the details of protocols used by different groups are different to some extent, the general steps should be similar which usually include consecutively pelleting the apoptotic bodies and cell debris, the MVs, and the exosomes, as shown in Figure 3. In most cases, samples are usually diluted by phosphate-buffered saline (PBS) before centrifugation to decrease their viscosity [27]. This dilution not only can increase the purity of EVs by decreasing the co-isolated contaminants, such as protein aggregates, but also can improve the efficiency of EV isolation since higher viscosity resulted in lower sedimentation efficiency [16,18,28]. After dilution, one or more centrifugation steps at 1000–3000× *g* are applied to remove dead cells and cell debris [15]. For example, a 30 min centrifugation at 2000× *g* can be used for viscous fluids according to one of the most cited protocols from Théry et al. [27].

Afterward, higher speed centrifugation, such as 10,000–20,000× *g*, typically follows to isolate MVs in the biofluids (Figure 3) [29,30]. The so-called ultracentrifugation at 100,000–200,000× *g* for hours is normally used to isolate exosomes from samples (Figure 3) [15,31]. Chutipongtanate et al. collected urinary MVs at a 20 min-centrifugation of 10,000× *g* before proceeding to prepare urinary exosomes at 100,000× *g* for 1 h [32]. Sun et al. also isolated MVs and exosomes from saliva samples by sequentially centrifuging at 10,000× or 20,000× *g* for 1 h and 100,000× or 125,000× *g* for 2.5 h, with 785 proteins identified from MVs and 910 proteins from exosomes [33]. Table 1 lists the details of centrifugation force and time from the selected EV studies for future reference. Their corresponding MS strategies and results are also included in Table 1. Rather than using common gel-based bottom-up proteomics, different methodologies on MS-based workflow were also developed and applied to EV studies as summarized in Table 1, such as different liquid chromatography (LC) fractionation methods, digestion strategies, and MS acquisition approaches, which will be discussed in Section 4. Many exosomes studies discarded the pellets resulted from 10,000–20,000× *g* before ultracentrifugation at 100,000–200,000× *g* (Table 1). However, Whitham et al. recently isolated EVs at 20,000× *g* for 1 h to study the exercise-induced EV proteome and found that a host of small-vesicle and exosomal markers, such as SDCBP, TSG101, PDCD6IP (ALIX), CD63, and CD9, identified in 20,000× *g*-derived EV lysates. Further quantitative studies revealed that no significant differences were observed in any EV markers between samples subjected to 20,000× or 100,000× *g* centrifugation. They claimed that a quantitative proteomic analysis of small-vesicle and exosomal protein cargo was possible with the 20,000× *g* centrifugation for 1 h rather than prolonged centrifugation at 100,000× *g* [34]. Besides, Kim et al. claimed that centrifugation at 40,000× *g* could provide comparable or improved results relative to ultracentrifugation at 110,000× *g* [35]. Those studies may imply that the purity of exosome samples yielded by dUC are obtained with the cost of exosome loss during centrifugation at 10,000–20,000× *g*.

The pellets of interest are usually washed once at the final steps by resuspension and centrifugationagain. It has been demonstrated that less washing can result in a higher EV yield, but also have more contaminants [36]. Therefore, the balance between yield and purity should be judged when adopting protocols. It is also worth noting that the efficiency of isolation is not only dependent on the viscosity of the samples, centrifugation force, and time, but also on rotor type since sedimentation path lengths are dependent on the type of rotors used and different distances from the rotational axis could result in differences in the g-force. Cvjetkovic et al. applied a 70 min centrifugation at 100,000× *g* for exosome isolation on three different rotors and found that the yield and purity of exosomes obtained were significantly different [37]. To address this issue, a web-calculator was developed by Livshits et al. to adjust the common dUC protocol to the “individual” dUC protocol [26]. Therefore, one should be aware that proper modifications are necessary when adopting dUC for different types of biofluids and laboratory settings in order to achieve optimal isolation.

dUC has been utilized to isolate MVs and exosomes from different types of biofluids, such as plasma, urine, saliva, breast milk, and semen, as listed in Table 1. But the EV pellets obtained from dUC are usually contaminated with some co-sediment high abundant components in the biofluids including lipoprotein participles, protein aggregates, and high abundant soluble proteins, which significantly affect the downstream MS analysis. To improve the purity of isolated EVs, density gradient (DG) flotation, such as the sucrose gradient or OptiPrep velocity gradient (iodixanol gradient), is developed and incorporated into the dUC protocol [15,50]. Although the density of MVs remains unclear, the density of exosomes is 1.13–1.19 g/mL [14]. Upon centrifugation, EVs migrate to the surrounding medium if their densities are same, resulting in further purification of the EVs from other contaminants. For example, the purified exosome pellets from dUC are resuspended into PBS and overlaid on a 30% sucrose cushion with centrifugation at 100,000× *g* [27]. The EV samples can be further fractionated by a step DG using a series of solutions with different density. Iwai et al. used a series of sucrose solutions with concentrations at 2.0, 1.6, 1.18, and 0.8 M and iodixanol solutions with concentration at 50%, 40%, 30%, and 20% to separately isolate exosomes from saliva and collect fractions from different densities [51]. A recent proteomic comparative study was performed to evaluate the dUC and DG and found that DG reduced the presence of co-isolated proteins aggregates and other membranous particles [52]. In comparison to the sucrose gradient, the OptiPrep velocity gradient is reported to perform better at removing some lipoproteins and preserving the size of the vesicles in the gradient [15]. One of the reasons is that the osmotic pressure of sucrose is higher than iodixanol, which could damage EVs in the samples [51].

Some additional strategies are also included in the dUC workflow to increase the purity of EVs for different types of biofluids. THP (also called uromodulin) is a highly abundant protein in urine and can form a polymeric network to trap exosomes during centrifugation at 10,000–20,000× *g*. To alleviate this effect and increase the yield of exosomes, incubation of the crude exosome pellets with dithiothreitol (DTT) or 3-[(3-cholamidopropyl)dimethylammonio]-1-propanesulfonic (CHAPS) were developed. DTT could denature THP, thus inhibiting aggregation and allowing THP to be removed from the supernatant. Moon et al. resuspended the 200,000× *g*-derived urinary pellets in the sucrose solution and incubated with 60 mg/mL DTT at 60 °C for 10 min before DG. A total of 1877 urinary exosome proteins were identified in MS^E^ analyses [42]. But one of the side effects caused by DTT is that exosomal protein remodeling as DTT is a strong reducing agent and may reduce the exosomal proteins, thus resulting in detrimental effects on their biological activity. Musante et al. used CHAPS which is a mild detergent and known to solubilize THP to replace DTT. They found that CHAPS did not affect vesicle morphology or exosomal marker distribution and preserved better biological activity. Further MS analysis revealed that 76.2% of proteins recovered by CHAPS were identified in those treated by DTT [53]. In addition, Barrachina et al. used KBr in a similar mechanism for plasma samples to reduce lipoproteins in EV samples by solubilizing them [54]. Alternative strategies to improve dUC can be achieved by combinational usage with other types of isolation methods, such as filter device or size exclusion chromatography (SEC). Those combinational methods not only can improve the purity of EVs, but also can dramatically reduce the overall processing time. Details will be presented in the following subsections.

### 2.3. Size-Based Isolation

Size-based isolation, such as filtration and size exclusion chromatography (SEC), is another type of isolation method, which can be used alone or with other methods to isolate EVs from biofluids. For filtration, samples are passed through a membrane with a specific pore size by centrifugation or pressure. Centrifugation-based filter devices have been reported to yield approximately three-fold greater EVs than that prepared by pressure-driven filter devices [55]. Filters made by different materials have been demonstrated as a fast and simple alternative to dUC. Merchant et al. applied a pore size 0.1 μm of commercially available VVLP (hydrophilized polyvinylidene difluoride) disc membranes to isolate urinary exosomes before MALDI (Matrix-assisted laser desorption/ionization) TOF analysis, and filtration of 50 mL urine samples was achieved within 15 min [56]. Musante et al. developed a “hydrostatic filtration dialysis” process to isolate urinary EVs. Urine samples were centrifuged at 2000× *g* before loaded onto a dialysis membrane with a molecular weight cut-off (MWCO) of 1000 kDa. They found that centrifugation at 2000× *g* allowed to remove the bulk of THP without losing exosomes. By using the dialysis membrane with MWCO of 1000 kDa, solvent, together with all the analytes below 1000 kDa were pushed through the mesh of the membrane due to the hydrostatic pressure of the urine. This method avoided the laborious and time-consuming steps of dUC, while the yield of EVs from this dialysis membrane was reported to outperform the dUC [57,58]. Sequential usage of different types of filters was also explored to isolate EVs. A three-step protocol was established based on sequential steps of dead-end pre-filtration, tangential flow filtration, and low-pressure track-etched membrane filtration. But this sequential filtration step was tested for cell culture, not for biofluids [59]. Instead of used alone, filtration is more commonly used with other types of methods for EV isolation, such as with dUC as a concentration/enrichment step with the aim to concentrate the samples and reduce the processing duration. For example, a 0.22 μm filter device is the most used filter device in EV studies to remove components with a diameter exceeding ca. 200 nm and as one of the processing steps in the dUC [16,60]. In the protocol of Théry et al., the pellets yielded by 2 h of centrifugation at 110,000× *g* were resuspended in PBS and passed through a 0.22 μm filter before another round of centrifugation at 110,000× *g* [27]. Shiromizu et al. further simplified the steps by initially using a 300× *g* centrifugation followed by a filtration step with a 0.22 µm filter to obtain the exosomes crude before a 30% sucrose DG in colorectal cancer biomarker studies [38]. The hydrostatic filtration dialysis can also be used as a pre-enrichment step for dUC to isolate urinary EVs [61].

Despite that the filtration is fast and has the capability of high throughput for EV isolation, the filters can be easily blocked resulted from trapping vesicles or other contaminant aggregates. SEC as another type of size-based isolation strategy has not been normally reported with this limitation posed by filtration [16]. For SEC, samples are loaded onto a column packed with heterogeneous polymeric beads, such as Sepharose, with diverse pore size. In general, the larger molecules are eluted earlier than the smaller ones since the smaller molecules can enter more pores than the larger ones, thus eluted later. Menezes-Neto et al. used SEC as a stand-alone methodology for isolation of EVs. They packed Sepharose CL-2B into a syringe and isolated exosomes from a 1 ml plasma after centrifugation at 500× *g* for 10 min. A total of 269 proteins were identified from the plasma of one healthy donor on an LTQ Orbitrap Velos mass spectrometer [62]. However, Karimi et al. also packed Sepharose CL-2B beads into a Telos solid phase extraction column and found that this SEC column failed to separate EVs from lipoproteins. Instead of using SEC alone, they overlaid a 6 mL plasma on top of an OptiPrep cushion and centrifuged at 178,000× *g* before SEC separation. The combinational usage of the density cushion and SEC reduced about 100-fold lipoprotein particles in the EV samples with 1187 proteins identified. [63]. SEC was also reported as an alternative step to replace the final step of dUC. Smolarz et al. used the SEC to isolate exosomes instead of ultracentrifugation at 100,000–200,000× *g*. Briefly, serum was centrifuged at 1000× *g* and 10,000× *g* for 10 and 30 min, respectively. The generated supernatant was filtrated using a 0.22 μm syringe filter unit before loading onto the micro-SEC column to isolate exosomes. A total of 267 proteins were identified by the downstream LC/MS analysis [64]. A commercial size-exclusion chromatography column, qEV, was also used to extract EVs from saliva and tears to study primary Sjögren’s syndrome [65]. One of the problems faced by SEC is the increased sample volume obtained after elution, resulting in an extra concentration step for the downstream EV analysis. Foers et al. compared ultracentrifugation and ultrafiltration for the concentration of the SEC eluent. They loaded 10,000× *g* supernatant of human synovial fluid into a HiPrep 26/60 Sephacryl S-500 HR prepacked gel filtration column. This column contains a hydrophilic, rigid allyl dextran/bisacrylamide matrix and allows for large sample volume input and small EV infiltration. SEC fractions were concentrated by either ultracentrifugation at 100,000× *g* for 90 min or passing an Amicon Ultra-15 100 kDa cellulose ultrafiltration device. They found ultrafiltration could avoid artifactual aggregation of EVs with contaminants, such as extracellular debris, which were typically observed in samples prepared by ultracentrifugation [66].

### 2.4. Precipitation-Based Isolation

Polymer precipitation-based isolation has the benefits of commercial availability and easy processing and is now widely applied to isolate EVs from the biofluids under many disease statuses, such as colorectal cancer, epithelial ovarian cancer, and rheumatoid arthritis [67,68,69]. This type of isolation method is initially used in viral studies by forming a polymer network to decrease the solubility of all components present in the sample [70]. The whole procedure includes mixing an appropriate volume of a polymer solution with samples and incubation. Then, the precipitated EVs are recovered by low-speed centrifugation. The polymer solution could be from a commercial kit, such as ExoQuick, Total Exosome Isolation, and ExoSpin, or home-made polyethylene glycol (PEG) solution [14]. Comparative studies have been conducted to evaluate the EVs isolated by different commercial kits in order to facilitate the choice of isolation methods. Ding et al. compared three commonly used commercial kits for EV isolation, including Total Exosome Isolation, ExoQuick, and RIBO Exosome Isolation Reagent. They found that the size of the majority of particles isolated by those kits was from 30–150 nm, while RIBO generated the highest particle yields. Further western blot (WB) results revealed that ExoQuick was the most efficient method by evaluating the marker proteins of CD63 and TSG101 [71]. Lobb et al. found that ExoSpin performed significantly better in avoiding co-isolation of contaminating proteins and yielded higher levels of EV markers compared to ExoQuick [55].

Although easy–to–use EV commercial kits are now widely used, home-made PEG has relative low-cost of EV preparation. Weng et al. added PEG into samples with a final concentration of 10% and incubated the samples at 4 °C for 2 h before recovery at centrifugation of 3000× *g*. Then a second-round of PEG precipitation was followed in order to improve the purity of EVs. The downstream MS analysis identified a total of 6299 protein groups from HeLa cell culture supernatant. Unfortunately, they did not test any biofluid sample in the study [72]. PEG has also been demonstrated to be used together with ultracentrifugation. Rider et al. purified the EVs resulted from one-round of PEG precipitation by further centrifugation at 100,000× *g* for 70 min [73]. Instead of isolating EVs by precipitation, aqueous two-phase systems (ATPSs) were proposed by Shin et al. They used a PEG/dextran ATPS to isolate EVs from the tumor interstitial fluid based on the mechanism that different kinds of particles are effectively partitioned to different phases in a short time. Their comparative studies showed that ATPSs could recovery about 70% of EVs from the EV protein mixtures, whereas the recovery for dUC and ExoQuick were about 16% and 40% [74]. But one should notice that EVs isolated by precipitation may be contaminated by polymer molecules, such as PEG, which is well-known for interfering in MS-based proteomic analysis. Therefore, it is necessary to remove those polymer molecules before MS analysis.

### 2.5. Affinity-Based Isolation

Apart from size and density, EVs share some common characteristics, like general protein composition and lipid bilayer structure. By utilizing those common characteristics, affinity-based isolation could achieve the isolation of EVs from complex biological samples. The main principle of affinity-based isolation is via the interaction between the surface markers of EVs with the antibody, molecules, or function group immobilized onto various carriers to separate EVs from the analyzed biofluids. Among those methods, immuno-based isolation is the most widely available and used method [15,75]. Some proteins have often been used as exosome-associated markers including the tetraspanin family (such as CD8, CD9, CD61, CD63, CD81, and CD82), cytoplasmic proteins (such as tubulin, actin, actin-binding proteins, annexins, and Rab proteins), and heat shock proteins (such as Hsp70, and Hsp90). Therefore, the antibodies against those common proteins coupled to different carriers have been utilized to isolate EVs [76,77,78]. Hildonen at el. isolated urinary exosomes from healthy subjects by immunocapture on magnetic beads. They coupled the antibody cocktail against CD8, CD61, and CD81 to magnetic beads. By digestions on beads in non-detergent containing buffer, they studied the outer membrane-associated proteins of exosomes and found 49 proteins associated or bound to membranes [76]. Antibody against tetraspanins was also shown to immobilize on highly porous monolithic silica microtips and applied to investigate lung cancer biomarker proteins on exosomes in serum samples. The subsequent MS analysis had identified 1369 proteins [77]. In addition to those common markers of EVs, immuno-based isolation was also explored to isolate the desired groups of EVs because the function of EVs appears to be determined by its specific protein content. For example, anti-EpCAM-coupled microbeads were employed to extract epithelial tumor-derived EVs from plasma since it has been demonstrated that exosomes from epithelial tumors express EpCAM (epithelial cell adhesion molecule) on their surface [78,79]. Tauro et al. isolated two distinct populations of exosomes released from organoids derived from the human colon carcinoma cell line LIM1863EVs, via sequential immunocapture using anti-A33- and anti-EpCAM-coupled magnetic beads [80].

In addition to antibodies, some EV-binding molecules, such as specific peptides including venceremin or Vn, and heparin, were also investigated to isolate EVs [14]. Vn, a novel class of peptides, which exhibit the specific affinity for heat shock proteins were selected for isolation of EVs from breast cancer [81]. Bijnsdorp et al. compared the urinary EVs isolated by Vn-96 and dUC and found that more than 85% of the proteins were identified both in EVs isolated by Vn and dUC. But the Vn96-peptide offered easier and time convenient methods in comparison with dUC [82]. Heparin is a highly sulfated glycosaminoglycan and has recently been used to isolate the EVs in which the surface contains the cell surface receptor, heparan sulfate proteoglycans. Balaj et al. incubated plasma with heparin-coated beads overnight and further processed the enriched samples by ultracentrifuging at 100,000× *g* for 90 min or a 100 kDa MWCO filter. The EVs isolated by heparin-affinity beads were detected to contain the EV marker of Alix and lower level of protein contamination [83].

Affinity for targeted proteins on the surface of EVs can be problematic for general EV studies since an unreliable analysis could be obtained due to the exclusion of EVs without targeted proteins.

Therefore, an affinity for the lipid membrane structures of EVs is utilized. Gao et al. recently adopted the TiO_2_ material, which is commonly used for the enrichment of phosphopeptides to isolate EVs. Through the interaction with the phosphate groups on the lipid bilayer of EVs, TiO_2_ can enrich EVs from serum within 5 min [84]. Tan et al. also focused on the membrane lipid as the target and used phospholipid-binding ligands to extract plasma EVs. Based on previous studies, EVs could be differentiated by their membrane phospholipid composition, specifically GM1 gangliosides and phosphatidylserines. They found two distinct groups of EVs by using cholera toxin B chain (CTB) and annexin V (AV), which, respectively, binds GM1 ganglioside and phosphatidylserine [85]. Nakai et al. developed a novel method for EV purification by using Tim4 proteins. Tim4 proteins can capture EVs via the specific interaction with the phosphatidylserine displayed on the surface of EVs and release the EVs by adding Ca^2+^ chelators. They claimed that the lower contaminations were found in the EV samples isolated by Tim4 proteins [86].

## 3. Comparative Studies for Isolation Methods of EVs

Among the isolation methods discussed above, it is generally thought that dUC is time-consuming. Filtration has the risk of stuck EVs in the membrane pores, while SEC is not ideal for large scale isolation. Although precipitation-based and immuno-based methods usually involve easy processing, the purity of EVs from precipitation is often problematic and affinity-based isolation is often considered as a good technique for isolation of sub-populations of EVs [16]. However, it is more reasonable to evaluate each isolation method based on the detailed protocol used and criteria of evaluation in each study. Otherwise, purity, efficiency, and reproducibility of different isolations could easily confound literature. For example, Kalra et al. performed a comparative evaluation of three exosome isolation techniques: dUC, anti-EpCAM conjugated microbeads, and OptiPrep DG. Their results suggested that the OptiPrep DG was superior in isolating pure exosomal populations by comparing the level of highly abundant plasma proteins which were detected by MS in the isolated plasma EV samples [79]. Those three methods were also compared by Greening et al. in a cell model. Based on the quantitative MS results for the identified exosome markers and proteins associated with EV biogenesis, trafficking, and release, anti-EpCAM was shown to be the most effective method to isolate exosomes [50]. Results from those two comparative studies can be explained by the differences in the sample types, details of protocols, and criteria of evaluation used in each study. Therefore, the selected studies for evaluation of different EV isolation methods are listed in Table 2 for better interpretation of each isolation. One thing to be mentioned is that the comparative studies listed in Table 2 also include the studies based on cell cultures, animals, and characterization of EVs by other methods, and are not just based on biofluid samples and analyses of MS.

As shown in Table 2, many studies have compared the EV isolation by different techniques; thus, according to different criteria. Different criteria were also applied, even if the same technique was used for assessment [55,88,92,94]. WB for EV marker proteins is one of the commonly used methods to compare the efficiency of EV isolation. But how many and which marker proteins should be chosen for the good comparison has not been well established. Lobb et al. provided a comparative analysis of four EV isolation techniques. dUC, ultrafiltration, SEC, OptiPrep DG, and precipitation (ExoQuick and ExoSpin) were used to isolate EVs from cell culture and plasma. By comparing the levels of exosomal markers of HSP70, Flotillin-1, and TSG 101 in WB, precipitation protocols provided the least pure preparations of EVs, whereas SEC isolation was comparable to DG purification of EVs [55]. In a similar way, Royo et al. tested the EV isolation of lectin-based purification, Exoquick, Total Exosome Isolation, and an in-house modified EV isolation procedure via WB of eight EV protein markers including CD9, CD10, CD63, TSG101, CD10, AIP1/Alix, AQP2, and FLT1. They observed that the levels of different EV marker proteins varied by different isolations and, thus, suggested that different methods isolated a different mixture of urinary EV marker proteins [92]. Evaluation of EV isolation by MS also lacks criteria to make a universal, comprehensive comparison. Rood et al. centrifugated the urine samples at 17,000× *g* for 15 min and then isolated the EVs by further centrifuging at 200,000× *g* for 110 min or filtering with 100 kDa Vivaspin 20 polyethersulfone nanomembrane concentrators. They found that either ultracentrifugation or ultrafiltration was difficult to isolate EVs from urine since highly abundant proteins, especially albumin and α-1-antitrypsin, were present in large amounts, which significantly limited the detection of MALDI-TOF. Additional SEC following ultracentrifugation was suggested to use in order to improve the purity of EVs [94]. Based on the gene ontology analysis for the identified proteins by MS, Davis et al. believed that dUC and SEC did not isolate equivalent EV population profiles [88]. Altogether, cautions should be taken when interpreting each EV isolation.

Rather than focus on the performance in yield or purity of each isolation, the functional activity of EVs was also reported to depend on the isolation method used [87,91]. Antounians et al. noticed that amniotic fluid stem cell-derived EVs isolated by dUC, precipitation (ExoQuick, Total Exosome Isolation Reagent, and Exo-PREP), and SEC (qEV column) had different effects on a model of damaged lung epithelium [91]. It suggests the necessity of evaluating the isolation methods within the content of biology.

## 4. MS Strategies Used in Proteomic Studies of Extracellular Vesicles

### 4.1. Sample Preparation and Separation

To date, proteomic studies of EVs are mainly conducted based on the bottom-up MS strategy. As shown in Figure 2, protein should be extracted from the isolated EVs and digested before MS analysis. For proteomic analysis, EV proteins are commonly extracted using the lysis buffer with detergent (such as sodium dodecyl sulfate (SDS)) or without detergent (such as 8 M urea). TRIzol reagent, which is often used in isolation of nucleic acid from EVs, has been recently reported to extract proteins from EVs. Joy et al. compared the EV protein extraction between Laemmli and TRIzol. Laemmli buffer typically contains 2% SDS, 10% glycerol in Tris-HCl with pH 6.8, which is an effective protein-extraction for EVs. They found that these two methods gave similar results in their ability to extract proteins and ~60% of proteins were identified in the samples prepared by both methods. However, they did not apply TRIzol reagent on any EV samples from biofluids [96]. Special extraction methods are also investigated to facilitate studies of sub-populations of proteins in the EVs, such as membrane proteins. Hu et al. optimized the Triton X-114 detergent partitioning protocol to target the analysis of membrane proteins of urinary EVs. Dried EV pellets were dissolved in 1% SDS containing lysis buffer for 1 h before adding 2.2% pre-condensed Triton X-114 buffer. A lower detergent phase, with an oily appearance, and an upper aqueous phase were formed when the temperature was above the clouding point of Triton X-114. Proteins in each phase were recovered by acetone precipitation before MS analysis. Most of the membrane proteins of urinary EVs were found in the detergent fraction [58].

**Figure 4:**
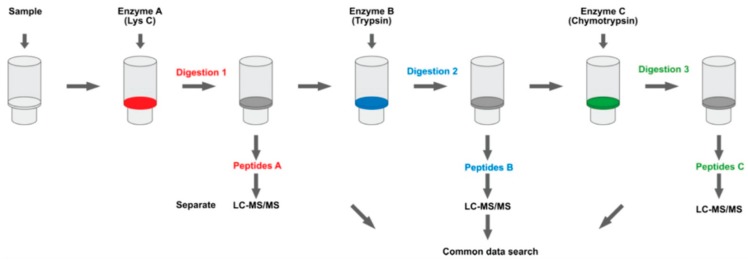
The schematic workflow for multi-enzyme digestion filter-aided sample preparation. This figure was adopted from Ref. [97].

As shown in Table 1, filter aided sample preparation (FASP) was utilized in some EV studies to achieve an easy process for buffer exchange and protein digestion [97]. In FASP, the extracted EV proteins are transferred into a molecular weight cut-off filter. This filter can retain most of the proteins on the membrane after simple centrifugation. Meanwhile, peptides can freely pass through the membrane during centrifugation. By using this kind of filter, the denaturing detergent-based buffer used for protein extraction can be easily changed to a digestion buffer, and the sample can be digested on the filter without extra transferring steps. FASP, with easy processing and minimal sample loss, has become the method of choice in many EV studies, especially in the limited amount of samples available [16]. Fel et al. improved the FASP by using multi-enzyme digestion to prepare EV samples obtained by precipitation. In their studies, serum samples from polycythemia vera patients were centrifuged at 2000× *g* for 30 min to remove cells and debris before incubation with the reagent from the Total Exosome Isolation kit. Afterward, the proteins were extracted from EVs and digested sequentially by Lys C, trypsin, and chymotrypsin in a Micron 30 kDa filter (Figure 4). A total of 706 proteins were identified with thirty-eight proteins showing significant differences in the patients’ group [97].

To perform in-depth proteomic analysis, additional separation before LC/MS analysis can be performed by either gel electrophoresis or liquid chromatography. Gel electrophoresis can effectively remove the most common contaminants in the samples according to the molecular weight of proteins, which could benefit the downstream MS analysis. Both Tsuno et al. and Xie et al. isolated EVs from serum using ExoQuick and separated the protein content through two-dimensional gel electrophoresis before MALDI-TOF analysis to study rheumatoid arthritis and coronary artery aneurysms, respectively [69,98]. Gel electrophoresis has also been applied to study EVs from urine, breast milk, and saliva [45,47,99]. Apart from separation based on gel electrophoresis, two-dimensional liquid chromatography (2D-LC) is utilized to analyze EV samples [30,38,39,40,100]. Antwi-Baffour et al. isolated MVs from the plasma of malaria patients and used a microcapillary strong cation exchange (SCX) column to fractionate the digested MVs samples. A total of 1729 proteins were identified in malaria samples, while only 234 proteins were identified in healthy control samples [30]. Their finding may imply that MVs in disease status could result in more protein identification than in healthy. Shiromizu et al. further simplified the fractionation of EV samples by using a C18-SCX Stage-tip. Using this strategy, they identified 702 proteins from the serum of colorectal cancer patients [38]. Instead of SCX as the first-dimensional separation, Lin et al. performed a high pH reverse phase chromatography to fractionate EVs from semen and study asthenozoospermia with 3699 protein identified by MS [40].

In addition to the typical proteomic studies, separation methods vary according to different studies, such as the studying of post-translational modifications of EV proteins. The electrostatic repulsion-hydrophilic interaction chromatography (ERLIC) was employed to facilitate the study of glycoproteins from EVs. Cheow et al. centrifuged plasma at 100,000× *g* for 2 h and 200,000× *g* for 18 h. They recovered a visible yellow suspension that was highly enriched in soluble glycoproteins and EVs. After protein extraction and digestion, an ERLIC column was used to simultaneously enrich secretory and EV-enriched glycoproteins and further fractionate the sample. A total of 127 plasma glycoproteins were identified with high confidence [101]. In order to study N-linked glycoproteomics of urinary exosomes, Saraswat et al. isolated urinary EVs by centrifugation at 200,000× *g* for 2 h and applied SNA affinity chromatography or SEC to enrich glycopeptides in the urinary EVs after tryptic digestion. In total, 126 *N*-glycopeptides from 51 *N*-glycosylation sites belonging to 37 glycoproteins were found [102].

### 4.2. MS Acquisition

During MS analysis, data-dependent acquisition (DDA) are normally used. Recently, data-independent acquisitions (DIA), such as SWATH (sequential window acquisition of all theoretical fragment ion), MS^E^, and multiplexed MS/MS, are used in EV studies to satisfy different purposes. Unlike DDA, DIA simultaneously fragments all precursor ions present in a wide isolation window. Braga-Lagache et al. analyzed MV proteins from plasma samples by both DDA and multiplexed DIA on a quadrupole orbitrap instrument. In each cycle of multiplexed DIA, data is usually acquired with one full MS scan followed by a series of MS2, such as ten MS2 scans. Each MS2 scan records all the fragment ions generated by precursor ions that are isolated from multiple different isolation windows with a fixed *m*/*z* range, such as isolated from three randomly combined 10 *m*/*z* isolation windows. A targeted approach is used to analyze the DIA data by using spectral libraries from formerly acquired fragment spectra with exact mass and retention time of precursors. They found that a multiplexed DIA approach only consumed one third of the DDA acquisition time when data was extracted by a targeted approach. Their results suggested that multiplexed DIA was a valuable alternative to DDA [103]. Moon et al. and Chutipongtanate et al. also applied DIA to analyze the protein content of EVs [32,42]. In the study of Moon et al., crude exosomes prepared by sucrose density ultracentrifugation were digested in-gel and analyzed by MS^E^ on a Waters Q-TOF mass spectrometer. In MS^E^, alternating low- and high-energy collision-induced dissociation are used. The low-energy scan is used to obtain precursor information, while the high-energy scan is to collect fragment ions. A total of 1877 urinary exosome proteins were identified from IgA nephropathy and thin basement membrane nephropathy patients [42]. Chutipongtanate et al. utilized SWATH to analyze urinary EV proteins. In SWATH, the mass range of interest is divided into several segments with a fixed *m*/*z* range, such as 25 *m*/*z*. Then, precursor ions within each segment are fragmented together until all the segments are analyzed. They achieve a label-free DIA quantitative analysis for EV and MV proteins with a curated spectral library of 1145 targets, suggesting their potential clinical use [32].

Quantitative MS based on label and label-free have been demonstrated to study various diseases, such as prostate cancer, asthenozoospermia and venous thrombosis [39,40,46,104]. Fujita et al. labeled the urinary EV proteins with isobaric tag for relative and absolute quantitation (iTRAQ). A total of 4710 proteins were identified by MS, including 3528 proteins quantified [39]. Lin et al. quantified seminal EV proteins with iTRAQ labeling and revealed 91 proteins with significant changes [40]. 2D-LC and tandem mass tag (TMT) were also used to quantitative analysis of EVs in HIV-infected alcohol drinkers and cigarette smokers through precipitation-based isolation [104]. Although stable isotope labeling by amino acids in cell culture (SILAC) cannot label EV proteins from human biofluids, a PROMIS-Quan method which based on SILAC quantification was developed in order to gain a comprehensive quantification for potential clinical EV protein analysis. In PROMIS-Quan, EV lysates were spiked with super-SILAC which was prepared from cell cultures and served as an internal standard. Then, the same set of super-SILAC mix was quantified relative to purified proteins of interest, with known absolute amounts. By this way, EV proteins can be quantified not only in large-scale but also retrospectively only relative to the same set of super-SILAC standard [29]. Quantitative MS is not only applied to the EV studies with the aim of biomarker discovery but also developed as an evaluation method to assess the EV isolation. Wang et al. established a multiple reaction monitoring (MRM) based method to assess the purity of EVs. MRM is often used for target quantitative analysis as a validation method for biomarkers reported in discovery MS analysis. They first generated ^15^N-labeled quantification concatamers (QconCATs) for a pattern of targeted EV proteins and abundant serum proteins (non-EV proteins or contaminants) as the internal standards for quantification of those proteins in MRM. QconCATs were artificial proteins composed of concatenated tryptic peptides from targeted proteins. The purity of EVs was then assessed by the quantitative results of the targeted EV proteins and abundant serum proteins in MRM [105]. They further expanded this method to separate EVs and lipoprotein particles by adding QconCAT for apolipoproteins into the previous MRM assay [106].

## 5. Conclusions

With a greater understanding of the roles of EVs in the regulation of physiological and pathological processes, an increased need to use that knowledge for diagnosis and therapy of diseases has emerged. To satisfy that increased need, establishing an EV isolation method that provides rapid, efficient, and high throughput isolation and enables assessment of the full spectrum of EVs is required. Unfortunately, the currently available isolation methods only partially meet the requirement. MS is a powerful tool for the characterization of the protein content of EVs, which is crucial to decipher the biological role of EVs and explore their potential use as diagnostic, monitoring, and therapeutic tools. Currently, the application of MS in EV studies is largely limited by the imperfections of EV isolation methods.

The increasing number of studies have pointed out the EV samples prepared by current isolation methods containing different sub-populations of EVs and contaminants from surroundings. Contaminants in the isolated EV samples may not only cover the signal of lower abundant EV proteins during MS analysis but also increase the difficulty of MS data analysis, since there is no current standard to clearly distinguish EV proteins from contaminants, especially the uncommon contaminants, in the MS-generated list. To address those problems, future improvements on EV isolation and MS analysis are urgently required.

## Figures and Tables

**Table 1:** Selected MS analysis for EVs obtained from centrifugation-based isolation.

Isolation	Proteomic Sample Preparation	Mass Spectrometry	Sample Origin	Number of Proteins	Year	Study
19,000× *g* for 120 min	2D-LC/MS: SCX as 1st dimensional	LTQ ion trap	plasma	1806 proteins	2017	[30]
Sucrose cushion at 100,000× *g* for 90 min	2D-LC/MS: C18-SCX stage-tip as 1st dimensional	Q-Exactive	serum	702 proteins	2017	[38]
100,000× *g* for 90 minincubation with DTT	iTRAQ 2D-LC/MS	LTQ-Orbitrap Velos Elite	urine	4710 proteins in total and 3528 proteins for quantification	2017	[39]
Sucrose cushion at 100,000× *g* for 90 min	iTRAQ 2D-LC/MS: high pH as 1st dimensional	Orbitrap Fusion Lumos	semen	3699 proteins in total	2018	[40]
110,000× *g* for 70 min	FASP	Q Exactive	serum	655 proteins	2018	[41]
10,000× *g*, 20 min for MVs and at 100,000× *g*, 1 h for exosomes	in-solution digestion	SWATH-MS TripleTof 5600+	urine	Targeted data analysis for 888 proteins	2018	[32]
Density ultracentrifugation at 270,000× *g*, 1 h and incubation with DTT	in-solution digestion	MS^E^	urine	1877 proteins	2011	[42]
100,000× *g* for 180 min	in-solution digestion	L Q-Exactive Orbitrap	umbilical cord blood	211 proteins	2015	[43]
200,000× *g*, 1 h and incubation with DTT	in-gel digestion	LTQ Orbitrap XL and LTQ Orbitrap Velos	urine	1989 proteins in total	2012	[44]
100,000× *g* for 90 min	in-solution digestion	LTQ Orbitrap Velos	saliva	381 proteins	2015	[45]
200,000× *g* for 90 min and incubation with KBr	iTRAQ LC off-line separation	MALDI * tandem mass spectrometry	plasma	not report	2010	[46]
Sucrose cushion at 192,000× *g* for 15–18 h	in-gel digestion	Q-Exactive	breast milk	1963 proteins	2016	[47]
20,000× *g* for 1 h for MVs	in-solution digestion	Q-Exactive/Plus	plasma	3294 proteins in 4 h LC/MS	2015	[29]
10,000 or 20,000× *g*, 1 h for MVs; 100,000 or 125,000× *g*, 2.5 h for exosomes	SDS-PAGE FASP	Q-Exactive	saliva	785 proteins for MVs; 910 proteins for exosomes	2018	[33]
20,000× *g*, 1 h for MVs; 100,000× *g*, 1 h for exosomes	in-solution digestion	LTQ-Orbitrap Velos Pro	plasma	9225 phosphopeptides in MVs; 1014 phosphopeptides in exosomes	2017	[48]
100,000× *g* for 70 min	in-gel digestion	LTQ-XL	CSF	91 proteins identified from control466 proteins identified from disease	2018	[49]

* MALDI: Matrix-assisted laser desorption/ionization.

**Table 2:** Selected comparative studies for EV isolation.

Isolation Methods	Characterization Techniques	Samples	Study
dUC, SEC	NTA, Dissociation-enhanced lanthanide fluorescence immunoassay, WB, TEM	rat plasma, cell culture	[87]
dUC, SEC	TEM, AFM, WB, MS	cell culture	[88]
Affinity-based (exoEasy kit) and SEC (qEV)	WB, TEM, NTA, lipid quantification kit, RNA quality	plasma	[89]
dUC and Commercial kit from Invitrogen, 101Bio, Wako and iZON	Dynamic Light Scattering, immunoblot analysis, qRT-PCR, MS, Cell Proliferation Assay	cell culture	[90]
dUC, precipitation (ExoQuick, Total Exosome Isolation Reafent, Exo-PREP) and SEC (qEV)	TEM, NTA, WB	cell culture	[91]
Lectin-based, Exoquick, Total exosome Isolation and in-house modified procedure	WB, Reverse transcriptase and qPCR, EM	urine	[92]
dUC, precipitation (ExoQuick, Total exosome isolation, PEG, Exo-spin), filtration (ExoMir)	NTA, Flow cytometry, WB, PCR,	serum	[93]
dUC, filtration (Stirred cell and Centricon), OptiPrep DG, ExoQuick, Exo-spin, SEC	Tunable resistive pulse sensing, EM, WB	cell culture and plasma	[55]
SEC and Exo-Spin	NTA, Flow cytometry, MS	plasma	[62]
dUC, anti-EpCAM, OptiPrep DG	MS, WB, TEM	plasma	[79]
Nanomembrane ultrafiltration, dUC and dUC-SEC	MS, TEM, WB	urine	[94]
dUC, anti-EpCAM, OptiPrep DG	TEM, CryoEM, MS	cell culture	[50]
Sucrose DG and ExoQuick	TEM, NTA, WB	serum	[95]

* EM: electron microscopy; TEM: transmission electron microscopy; NTA: nanoparticle tracking analysis AFM: atomic force microscopy; WB: western blot.
